# Effects of dexamethasone on progesterone and estrogen profiles and uterine progesterone receptor localization during pregnancy in Sahel goat in Semi-Arid region

**DOI:** 10.1186/s40781-017-0137-5

**Published:** 2017-05-26

**Authors:** Dauda Yahi, Nicholas Adetayo Ojo, Gideon Dauda Mshelia

**Affiliations:** 1 Department of Veterinary Physiology and Biochemistry, Faculty of Veterinary Medicine, University of Maiduguri, P. M. B, Maiduguri, 1069 Nigeria; 2 Department of Theriogenology, Faculty of Veterinary Medicine, University of Maiduguri, P. M. B, Maiduguri, 1069 Nigeria

**Keywords:** Dexamethasone, Estrogen, Pregnancy, Progesterone, Sahel goat, Uterus

## Abstract

**Background:**

Despite the widespread use of dexamethasone in veterinary and human medicine, it is reported to cause some severe pregnancy related side effects like abortion in some animals. The mechanism of the response is not clear but seems to be related to interspecies and/or breed difference in response which may involve alterations in the concentrations of some reproductive hormones.

**Methods:**

Twenty Sahel goats comprising 18 does and 2 bucks were used for this study. Pregnancies were achieved by natural mating after synchronization. Repeated dexamethasone injections were given at 0.25 mg/kg body weight. Blood samples were collected biweekly for hormonal assay. Uterine biopsies were harvested at days 28 and day 78 of gestation through caesarean section for immunohistochemical analysis using 3 pregnant does randomly selected from each group at each instant. Data were expressed as Means ± Standard Deviations and analyzed using statistical soft ware package, GraphPad Instant, version 3.0 (2003) and progesterone receptor (PR) were scored semi-quantitatively.

**Results:**

Dexamethasone treatments had no significant (*p* > 0.05) effect on progesterone and estrogen concentrations in pregnant Sahel goats but up regulated PR from 2+ to 3+ in second trimester.

**Conclusion:**

As dexamethasone adverse effect on placenta is an established fact, the lack of effect on progesterone level in this study may be due to the fact that unlike other species whose progesterone production during pregnancy is placenta – dependent, in goats is corpus luteum - dependent. Consequently dexamethasone adverse effect on placenta reported in literatures did not influence progesterone levels during pregnancy in Sahel goat. The up regulation of progesterone receptor (PR) in Sahel goat gravid uterus is a beneficial effects and that dexamethasone can safely be used in corpus luteum – dependent progesterone secreting pregnant animal species like Sahel goat and camel. Therefore source of progesterone secretions during pregnancy should be considered in clinical application of dexamethasone in pregnancy.

## Background

Progesterone and estrogen are chemically classified as steroids and are regarded as the two main reproductive steroidal hormones in mammals [1]. Progesterone has a central role in reproduction, being involved in maintenance of pregnancy. During pregnancy, progesterone is mainly produced by corpus luteum (C.L) and placenta, and to lesser extent, in the adrenal cortex [2–5]. Estrogens are usually produced by the mammalian ovary, corpus luteum or placenta and may be conjugated [6, 7]. Estrogen is also known to be produced by both maternal and foetal adrenal glands during pregnancy in addition to corpus luteum and placenta. During foetal adrenal steroidogenesis, foetal adrenal cortex is the principal source of placental estrogen precursors [8–10]. Cholesterol is converted in the foetal adrenal glands to dehydroepiandrosterone (DHEAS). The DHEAS is converted to estradiol or estrogen by the placenta [8–10]. Recent findings suggest that progesterone action to maintain uterine quiescence may be indirect by inhibiting expression of contractile genes within the uterus and cervix and blocking the production of chemokines that promote chemotaxis of immune cells [11]. Also progesterone is known to inhibit prostaglandin synthesis and activity in pregnant subjects [12] and consequently decreases myometral contractility. This inhibition is mediated by a number of pathways that include blocking prostaglandin action, decreasing prostaglandin synthesis, and increasing its rate of inactivation [12]. A fall in progesterone levels during pregnancy is associated with increased prostaglandin synthetase activity and prostaglandin F_2_α production, and this predisposes to abortion [1, 13, 14].

As the effects of progesterone are mediated by its nuclear receptor [15], the regulation of PR genes is critical to progesterone responsiveness of the uterus and thus the maintenance of uterine quiescence during pregnancy. Therefore progesterone and its receptor (PR) are critical components of uterine physiology in pregnancy.

One conserved function of steroid hormone receptors is that they autoregulate the expression of their own genes [16]. In general, hormone receptors are regulated both by their own ligand (homologous regulation) and by other regulatory molecules (heterologous regulation). Endogenous glucocorticoids are known to be involved in the heterologous up-regulation of several hormone receptors [17].

Dexamethasone being a synthetic glucocorticoid may have similar role in pregnancy. Dexamethasone is commonly used to treat and manage several diseases and other medical conditions in both animals and humans. These include pregnancy related and metabolic diseases such as ketosis, pregnancy toxaemia and mastitis [18, 19], prenatal foetal lung development and maturation, management of neonatal diseases [20, 21]. However, since there is no single drug that produces just a single effect without being accompanied with other undesirable effects, dexamethasone is no exception. The drug has been reported and observed to cause abortions in some breeds/species of animals like cattle, sheep and dog [22, 23]. These adverse effects of dexamethasone may be related to alterations in the normal concentrations of progesterone and estrogen and their receptors during pregnancy. Nonetheless different species or breeds do not always respond to medicines in the same way due to differences in anatomy, metabolism and inherent pharmacokinetics. Hence there is paucity of information on the effects of dexamethasone in Sahel goats during pregnancy. The objective of the present study therefore was to determine effects of dexamethasone on serum progesterone and estrogen profiles and progesterone receptors concentrations in Sahel goats during gestation.

## Methods

We followed the methods of Yahi et al. [24] and Yahi et al. [25] in our methodology.

Twenty clinically healthy adult Sahel goats comprising 18 does and 2 bucks were used for this study. The animals were purchased from Kasuwa Shanu livestock market and private farms in Maiduguri Metropolis. The ages of the does ranged between 2 and 3 years and the bucks ranged between 2½ and 3 years based on dentition and breeding history [26]. The does weighed between 25 to 28 kg and the bucks weighed between 25 and 32 kg. The Body Condition score (BCS) between 3.0 and 3.5 was maintained during the period of the experiment in all the animals. They were managed intensively in the University of Maiduguri Livestock Farm and were acclimatized for four weeks before the commencement of the experiment. The males and the females were initially kept in different pens until the time of service. The feed rations consisted of wheat offal, beans husks and hay from groundnut leaves. Mineralized salt licks and water were provided ad libitum. During the stabilization period, the animals were treated with oxytetracycline LA (Introxin-200®, Interchiemie, Venray, Holland) at 20 mg/kg body weight and ivermectin (paramectin®, Pharma Swede, Egypt) at 200 μg/kg body weight.

### Estrus synchronization

All the females were synchronized at the end of the acclimatization period using cloprostenol (Estrumate®, Schering Trough Animal, Germany) at 250 μg/kg given intramuscularly 11 - day interval as reported by Akusu and Egbunike [27]. They were teased with apronned males daily and all the females that came into estrus after the second treatment were allowed to be served naturally by the males. Days of estrus were recorded and considered as day 0 of the gestation.

After successful synchronization and fertile mating, the animals were randomly separated into 2 groups of 9 each. Accordingly, the groups were as follows: Dexamethasone treated goat (DTG), and Non dexamethasone treated goat (NDG) (Control).

### Treatments

The animals in the dexamethasone treated group were treated with dexamethasone (Dexaphan®, Pharma Pharmaceuticals, Swede-Egypt) injection given intramuscularly at 0.25 mg/kg body weight on days 1, 3 and 5 during first trimester; day 51, 53 and 55 during second trimester, and day 101, 103 and 105 during the third trimester. They were observed for possible clinical changes throughout the period of the experimentation. Pregnancies were later confirmed by ultrasonograhic examination using Draminski Ultrasound Pregnancy Detector (UPD-PD032013EX-1.2, Draminsky Agricultural Engineering Co. Inc., Owocowa-Olsztyn, Poland).

### Blood sample collection and analysis

Five ml of fasting blood samples were collected from day 0 and thereafter on biweekly basis in each animal through the jugular vein on the same day with minimal excitement. The samples of were placed in sterile sample bottles without anticoagulant and the blood were allowed to clot and centrifuged at 3000 rpm for 5 min. The sera were harvested and stored at −20 °C until assayed for progesterone and estrogen assay using standard goats ELISA kits (BlueGene, BioTech Inc., Shanghai, China).

### Immunohistochemistry

Biopsies of the uterus were harvested at days 28 and day 78 of the first trimester and second trimester of gestation respectively through caesarean section using three (3) pregnant does randomly selected from each group at each instant. Three (3) does from each group were allowed full term with normal delivery. The samples were fixed in 10% neutral buffered formalin for immunohistochemistry and progesterone receptor localization while the foetuses harvested were used for other investigations. Immunohistochemistry was carried out on paraffin- embedded sections of the uterine specimens using mouse monoclonal antibody for progesterone receptor and used according to standard protocols [28]. Tissue molds were cut into sections of 5 μm thick by microtome machine and placed in water bath and warmed and fixed onto Poly-Lysine coated pre-cleaned immunohistochemistry tissue slides (1′×3′×10 mm) and sections were dried and processed using standard immunohistochemical staining protocols as described by the manufacturers. Mouse monoclonal primary antibody (RE-7102) to the progesterone receptor obtained from the Novocastra™, Germany, was used for progesterone receptor localization. Blocking serum, biotinylated secondary antibody against mouse IgG and avidin-biotin complex was obtained from the Ultra Vision-Thermo Fisher scientific Co. Inc. kit (TA-060-PBQ) and used according to the manufacturers’ instructions. The processed slides were then viewed under light microscope. The intensity and percentage of each stained cells of the immunohistochemistry staining for the progesterone receptors were analyzed using light microscope (Multiple Headed Microscope; DESC-LN-0100-MG001, Vamed Engineering, UK). Microphotographs were taken using Canon IXUS Camera, pixel: 16.5 (China). The stained sections were evaluated and scored semi quantitatively both in terms of percentage or number of stained cells and staining intensity as described by Diest et al. [29]. This involved systematic random sampling of fields of vision. Negative staining, weak, moderate, strong and very strong positive staining was scored visually on a scale from 0 to 4 respectively.

### Statistical analyses

Data collected were expressed as Means ± Standard Deviations (S.D). The Significant differences between the dexamethasone treated and non dexamethasone treated groups were compared using Student’s t – test. Significant differences were considered at *p* < 0.05. Computer statistical software package, GraphPad InStat® [30] was used for the analyses.

## Results

Changes in concentration of progesterone and estrogen in pregnant Sahel goats are presented in Table 1. There was no statistically significant (*p* > 0.05) variation in progesterone and estrogen concentrations between dexamethasone treated group and the control even though the concentrations of both progesterone and estrogen were decreased in dexamethasone treated goats compared to control. However, the immunohistochemical evaluation of the gravid uteri showed that dexamethasone intensely up regulated the concentration of progesterone receptor (PR) in the Sahel goat uteri compared to control groups (Plates I, II). Staining intensity for uterine progesterone receptors was observed to be strongly positive (3+) in dexamethasone treated pregnant Sahel goats but weaker (2+) in the control group. Immunoreactivity was generally localized in the nuclei of the positive cells. There were abundant progesterone receptors nuclear staining of both the glandular cells and the majority of stromal cells and myometrial cells in both groups. However, more intense progesterone receptor concentrations were observed in the treatment group compared to control. Intense progesterone receptor concentrations were mostly expressed in the glandular epithelia of the endometrial glands compared to the stromal and myometrial cells. During first trimester (day 28), the staining intensity for progesterone receptor in the control group was moderate (2+) in all parts of the uterus. In dexamethasone treated group, staining intensity for uterine progesterone receptors was observed to be strongly positive (3+) (Plate Ib) as opposed to weaker intensity (2+) in the respective control group (Plate Ia). The expression of the progesterone receptors staining was positive (3+) in the glandular epithelia cells, moderate positive (2+) staining in the stromal and luminal cells. In second trimester (day 78), the staining characteristics in the control group uterus did not change but remained at the level of moderate (2+) staining in all parts of the uterus. Similarly, in the dexamethasone treated group, the staining intensity pattern of positive (3+) in the glandular epithelia cells, moderate positive (2+) staining in the stromal and luminal cells was maintained.

**Table 1:** Effects of dexamethasone on serum progesterone and estrogen concentrations in pregnant Sahel goat

Gestation Period (Days)	Progesterone(ng/ml)		Estrogen(pg/ml)	
	DTG (*N* = 9)	NDG (*N* = 9)	DTG (*N* = 9)	NDG (*N* = 9)
0	0.10 ± 0.01	0.10 ± 0.01	7.32 ± 0.25	7.30 ± 0.25
14	6.33 ± 0.45	6.32 ± 0.50	2.27 ± 0.29	2.28 ± 0.30
28	7.22 ± 0.30	7.24 ± 0.30	2.43 ± 0.50	2.44 ± 0.38
42	7.26 ± 0.71	7.28 ± 0.55	28.85 ± 0.44	29.16 ± 0.47
56	11.17 ± 0.54	11.18 ± 0.49	86.71 ± 0.55	87.0 ± 0.20
70	11.32 ± 0.26	11.31 ± 0.23	90.9 ± 0.60	91.3 ± 0.20
84	12.25 ± 0.30	12.27 ± 0.30	112.33 ± 1.20	111.35 ± 1.19
98	11.64 ± 0.50	11.64 ± 0.50	117.43 ± 1.22	116.45 ± 1.20
112	11.60 ± 0.51	11.60 ± 0.51	119.30 ± 3.25	120.33 ± 3.10
126	9.37 ± 0.0.25	9.37 ± 0.0.25	210.46 ± 1.31	209.48 ± 1.30
140	8.12 ± 0.36	8.12 ± 0.36	223.90 ± 2.33	225.51 ± 1.21

*DTG* Dexamethasone treated, *NDG* Non dexamethasone treated (Control), *N* Sample size. There was no significant (*p* > 0.05) variation in progesterone and estrogen concentrations between dexamethasone treated group and the control group

**Plate I:**
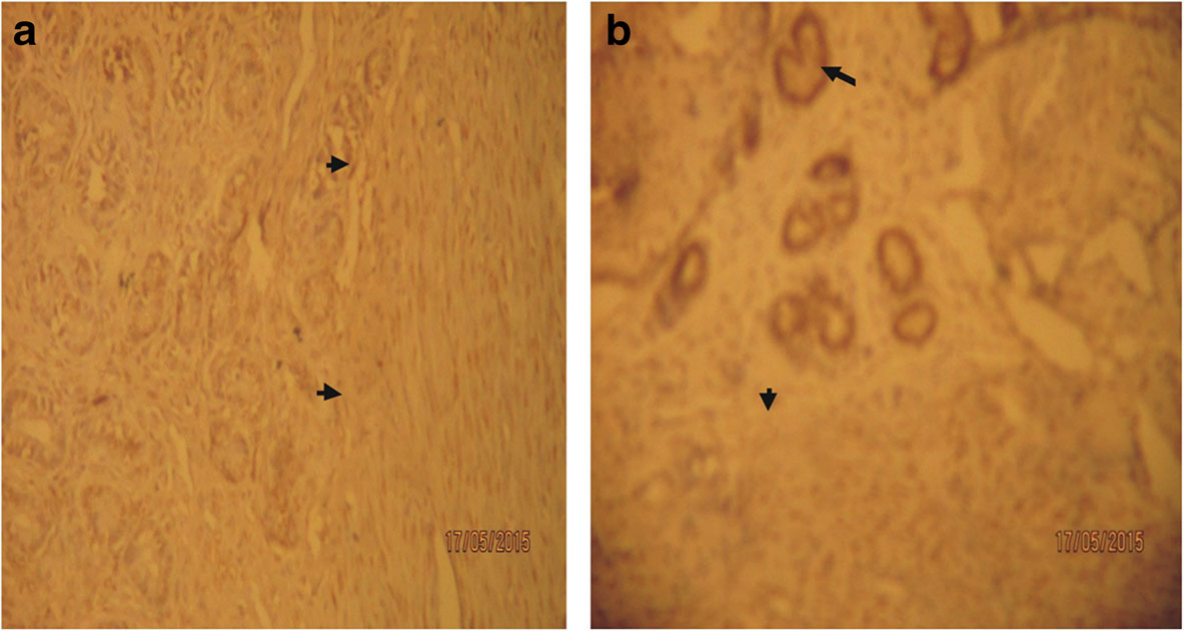
Progesterone receptor staining of Sahel goat uteri during first trimester (day 28 of gestation). *Arrow heads* indicate moderate positive progesterone receptor (PR) staining (2+) and eosinophilic cells while *arrow* indicates strong positive staining (3+) for progesterone receptors (PR) and endometrial lymphocyte. Plate I**a** (Control goat): Progesterone receptor staining indicated moderate positive staining (2+) in the stromal, luminal and glandular epithelia cells (IHC × 100). Plate I**b** (Dexamethasone treated goat): Progesterone receptor staining showed strong prositive staining (3+) in the glandular epithelia cells, moderate positive staining (2+) in the stromal and luminal epithelia cells (IHC × 100)

**Plate II:**
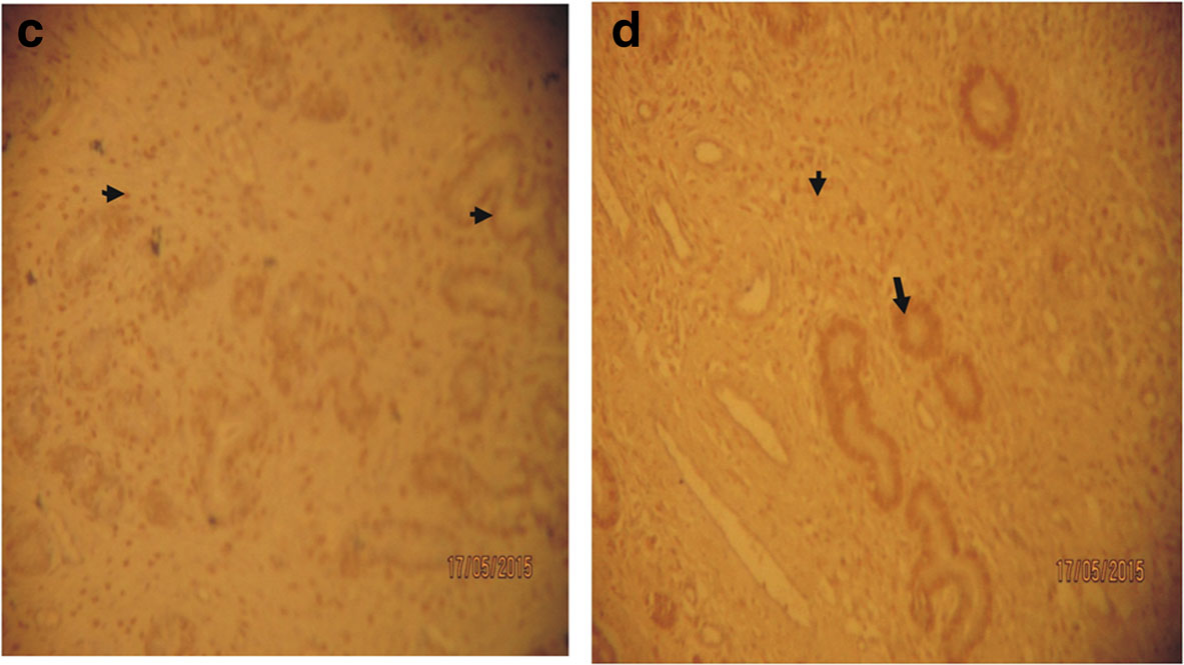
Progesterone receptor staining of Sahel goat uteri at day 78 of gestation (second trimester). *Arrowheads* indicate moderate positive progesterone receptor (PR) staining (2+) and eosinophilic cells while *arrow* indicates strong positive staining (3+) for progesterone receptors (PR) and endometrial lymphocyte. Plate II**c** (Control goat): Progesterone receptor staining showed moderate positive staining (2+) in the luminal, glandular and stromal epithelial cells (IHC × 100). Plate II**d** (Dexamethasone treated goat): Progesterone receptor staining showed moderate positive staining (2+) in the stromal and luminal epithelial cells and strong positive (3+) in the glandular epithelia cells (IHC × 100). Legend: PR: Progesterone receptor; IHC: Immunohistochemistry

The six does, three from each group had normal parturition. Two does from the control group parturited on day 147 and one on day 148 respectively. The mean gestation length was 147.5 ± 1.71 days. The mean birth weight was 1.8 ± 0.4 kg and the mean placental weight at birth was 647 ± 0.31 g. All the dexamethasone treated does parturited on day 148. The mean birth weight and mean placental weight at birth in dexamethasone treated does were 1.7 ± 0.40 kg and 494 ± 0.30 g respectively (Table 2). The results suggest that maternal dexamethasone treatment did not affect gestational length (148.0 days) as compared with the control groups (147.5 ± 1.71 days), but causes significant decrease in birth weights (1.7 ± 0.40 g) and placental weights (494 ± 0.30 g) in the Sahel goats as compared with the control group (1.8 ± 0.4 g and 647 ± 0.31 g respectively) (Table 2).

**Table 2:** Effects of dexamethasone on gestational lengths, birth and placental weights in Sahel goat

Parameters	Groups	
	DTG	NDG
Gestational length (days)	148.0 ± 0.0	147.5 ± 1.71
Birth Weight (kg)	17.0 ± 0.40^a^	1.8 ± 0.40
Placental weight (g)	494 ± 0.30^a^	647 ± 0.31

*DTG* Dexamethasone treated, *NDG* Non dexamethasone treated (Control)

^a^Significant decrease compared to control

## Discussion

As mentioned in the results above, this study indicated that the progesterone and estrogen concentrations were not significantly affected by dexamethasone treatment during pregnancy in goats. The observation in this study is similar to that of Ohrlander et al. [31] who reported that dexamethasone administered to induce foetal lung maturation in human did not alter the serum concentrations of progesterone, but differs from that of Ahmadabad et al. [32] who reported decrease progesterone levels in pregnant mice treated with dexamethasone. The differences observed could be attributed to species differences with respect to source of progesterone secretions during pregnancy. Progesterone is mainly produced by corpora lutea (C.L) and placenta, and to lesser extent, the adrenal cortex during gestation in most mammals [2, 4]. Unlike mice, in goats, progesterone production during gestation is mainly corpus luteum - dependent with little or no contribution from placenta [33–35]. Despite the widespread clinical use of dexamethasone, it has been reported to cause adverse effects on placenta which include decreased placental weight and placental efficiency in some animal models as well as humans [22, 23, 36–38]. Therefore, since in goats, progesterone is mainly produced by C.L during pregnancy [33–35], possible adverse effects on placental progesterone production is of little significance in goats. This may account for the lack of effect on progesterone secretion despite dexamethasone placental adverse effects during gestation in goats.

On the other hand the lack of effect of dexamethasone on estrogen level during pregnancy in the Sahel goats is in contrast to earlier report by Ylikorkala et al. [39] and that of Ahmadabad et al. [32] who reported decrease progesterone and estrogen levels in pregnant mice treated with dexamethasone. The lack of effect of dexamethasone on estrogen concentration in this study suggests that dexamethasone does not have negative effects on estrogen and estrogen precursor producing structures like ovaries and adrenal glands [40–42].

The up regulation of the PR in the goat uterus by dexamethasone observed in this study could be one of the beneficial effects of dexamethasone in an attempt to increase progesterone sensitivity. McDonald et al. [17] reported that glucocorticoids are involved in the heterologous up-regulation of several hormone receptors. The mechanism is probably through regulation of receptor mRNA levels by influencing increase in PR mRNA levels and gene transcription as reported by Kraus and Katzenellenbogen [43] in rats and Leavitt et al. [44] in humans. Therefore, dexamethasone probably stimulates transcriptional activity of PR and increases total PR expression in the uterus. Also it is possible that the observed increase in PR immunoreactivity could be due to an increase in the ligand-independent expression of PR by insulin – like growth factor (IGF-I) [45] which may be mediated by dexamethasone. In this context dexamethasone is known to increase metabolism [46]. The decreased neonatal birth weight may be due to decreased utero-placental exchange or perfusion probably as a consequence of decreased placental weight or function.

## Conclusion

This study confirms previous findings that antenatal dexamethasone retards placental growth. While dexamethasone adverse effect on placenta is an established fact, the lack of effect on progesterone level in this study was due to the fact that unlike other species whose progesterone production and secretion during pregnancy is placenta – dependent, in goats it is corpus luteum - dependent. Consequently dexamethasone adverse effects on placenta, as observed in this study and reported in other literatures, does not influence progesterone levels during pregnancy in Sahel goat; instead dexamethasone up regulated PR. The intense progesterone receptor concentrations were mostly expressed in the glandular epithelia of the endometrial glands compared to the stromal and myometrial cells. The up regulation of PR in Sahel goat gravid uterus is a beneficial effects and that dexamethasone can safely be used in corpus luteum – dependent progesterone secreting pregnant animal species like Sahel goat and camel. However, in placenta – dependent progesterone secreting pregnant subjects, dexamethasone should be used with caution.

## Abbreviations

BCS: Body Condition score
C.L: Corpus luteum
DHEAS: Dehydroepiandrosterone
DTG: Dexamethasone Treated Goat
ELISA: Enzyme Linked Immuno-Sorbent Assay
IGF-I: Insulin – Like Growth Factor
kg: Kilogram
LA: Long Acting
mg: Milligram
N: Sample size
NDG: Non Dexamethasone Treated Goat
p: *p* value
PR: progesterone receptor
UPD: Ultrasound Pregnancy Detector
μg: Microgram
